# MODalyseR—a novel software for inference of disease module hub regulators identified a putative multiple sclerosis regulator supported by independent eQTL data

**DOI:** 10.1093/bioadv/vbac006

**Published:** 2022-01-25

**Authors:** Hendrik A de Weerd, Julia Åkesson, Dimitri Guala, Mika Gustafsson, Zelmina Lubovac-Pilav

**Affiliations:** 1 School of Bioscience, Systems Biology Research Center, University of Skövde, Skövde 541 45, Sweden; 2 Department of Physics, Chemistry and Biology, Linköping University, Linköping 581 83, Sweden; 3 Department of Biochemistry and Biophysics, Stockholm University, Solna 17121, Sweden; 4 Merck AB, Solna 16970, Sweden

## Abstract

**Motivation:**

Network-based disease modules have proven to be a powerful concept for extracting knowledge about disease mechanisms, predicting for example disease risk factors and side effects of treatments. Plenty of tools exist for the purpose of module inference, but less effort has been put on simultaneously utilizing knowledge about regulatory mechanisms for predicting disease module hub regulators.

**Results:**

We developed MODalyseR, a novel software for identifying disease module regulators and reducing modules to the most disease-associated genes. This pipeline integrates and extends previously published software packages MODifieR and ComHub and hereby provides a user-friendly network medicine framework combining the concepts of disease modules and hub regulators for precise disease gene identification from transcriptomics data. To demonstrate the usability of the tool, we designed a case study for multiple sclerosis that revealed IKZF1 as a promising hub regulator, which was supported by independent ChIP-seq data.

**Availability and implementation:**

MODalyseR is available as a Docker image at https://hub.docker.com/r/ddeweerd/modalyser with user guide and installation instructions found at https://gustafsson-lab.gitlab.io/MODalyseR/.

**Supplementary information:**

Supplementary data are available at *Bioinformatics Advances* online.

## 1 Introduction

Research in network medicine has to a high extend focused on developing new algorithms for inference of disease modules (Bader and Hogue, 2003; Bruhn *et al.*, 2014; Ghiassian *et al.*, 2015; Gustafsson *et al.*, 2014a; Langfelder and Horvath, 2008, 2012; Vlaic *et al.*, 2018) and benchmarking these (Badam *et al.*, 2021; Choobdar *et al.*, 2019; de Weerd *et al.*, 2020) to come up with ‘gold standard’ approaches in this field. We recently developed MODifieR (de Weerd *et al.*, 2020) to integrate the result of eight different module detection methods and evaluate their performance. However, it remains challenging to translate these modules into clinical applications by for example utilizing them to uncover biomarkers associated with diseases. A potential reason for this include that modules often are large and non-specific, often covering plenty of genes, which could not be associated directly using the omic that was used for detection. We and others have used modules as a first coarse-graining basis for extracting most relevant disease-associated genes by integrating knowledge about regulatory mechanisms inside the modules that could potentially explain the driving force behind the disease processes or the effect of a certain treatment. The concept of forming such modules by integrating variables of importance for pathogenesis could also serve as a ‘digital twin’ model to individualize patient treatment bringing us closer to precision medicine (Björnsson *et al.*, 2019; Cirillo and Valencia, 2019; Gligorijević *et al.*, 2016; Malod-Dognin *et al.*, 2018).

Another type of popular network tool that has been used successfully in systems medicine is hub analysis, which aims at identifying a minimal set of upstream core regulators (often <10) that are significantly enriched regulating a gene set (Gustafsson *et al.*, 2015; Lefebvre *et al.*, 2010; Liu *et al.*, 2019). Hub analysis has been hampered by similar problems as modules, i.e. lack of agreement in golden standards that has hampered model comparisons and development. This led us to recently develop ComHub for robust prediction of hubs in gene regulatory networks combining the prediction of six different methods (Åkesson *et al.*, 2021).

Modules and upstream hubs are rarely combined except for some individual methods like WGCNA (Langfelder and Horvath, 2008, 2012). We therefore developed MODalyseR, which combines MODifieR, and ComHub in a systems medicine pipeline tool. To increase its usability for a great variety of researchers, we have included it as a R shiny web application in a docker image to run on all operating systems, with the option to export computationally demanding methods as an automated nextflow (Di Tommaso *et al.*, 2017) pipeline to efficiently run on computer clusters. As a showcase, we applied MODalyseR to white matter lesions from patients with multiple sclerosis (MS).

In MS parts of the white matter of the central nervous system is damaged by immune cells. MS is one of the most common causes of disability in young adults (MSIF, 2020), and is a highly multi-factorial autoimmune disease with more than 200 associated interacting single-nucleotide polymorphisms (SNP) and environmentally associated risk factors (Olsson *et al.*, 2017). These factors have in several studies shown to co-localize within MS modules of the protein interaction network (Gustafsson *et al.*, 2014b; Hellberg *et al.*, 2016; Menche *et al.*, 2015), which further also are upstream regulated through hub transcription factors (Gustafsson *et al.*, 2015).

MODalyseR first identified modules of 1200 proteins that were independently confirmed MS enriched. Through our hub enrichment analysis the modules were narrowed down to 40 proteins and IKZF1 identified as an upstream hub TF, which was again supported by independent data. We therefore believe MODalyseR to be an important contribution to reproducible research and standardized network analysis for clinicians and bioinformatic support services.

## 2 Methods

### 2.1 Implementation of application

MODalyseR is a standalone interactive R Shiny web application implemented in the golem framework (Guyader *et al.*, 2020) and shipped as a Docker container that can be run on a local machine. The frontend of the application is an interactive reactive website accessible in a web browser on the localhost. The backend is implemented in the R programming language and consists of the functions implemented in the MODifieR R package and ComHub Python library with in addition functions to integrate the result of each of the packages into a single module. Persistent storage of input data and results between user sessions is provided by an SQLite database integrated in the application. To interface with the database, the RSQLite R package (Müller *et al.*, 2020) is used. Cytoscape 3.8.2 (Shannon *et al.*, 2003) is included in the Docker image and the RcY3 (Ono *et al.*, 2015) package is used to communicate between MODalyseR and Cytoscape. For computationally demanding methods and datasets a self-contained nextflow script that automatically downloads a singularity image (Kurtzer *et al.*, 2017) is provided to execute the methods on an external computing source, for example a High Performance Computer cluster. The output of this nextflow analysis can then be uploaded to MODalyseR.

### 2.2 Pipeline

**Fig. 1.**
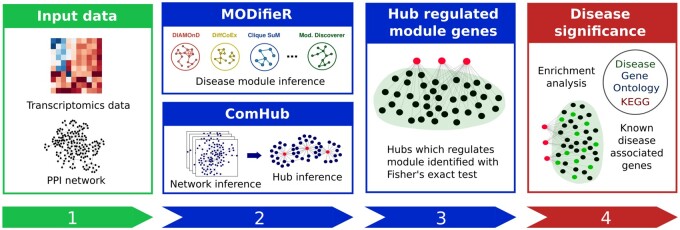
The MODalyseR pipeline. (1) Transcriptomics data and a PPI network are used as input. (2) Disease modules are inferred with MODifieR. Simultaneously, ComHub is used to infer hub regulators using the predictions by a compendium of network inference methods. (3) Inferred disease modules are filtered on hub regulation resulting in a smaller module with stronger disease association. (4) The disease significance of the resulting module is analyzed using enrichment analysis

MODalyseR implements a pipeline for disease module analysis (Fig. 1). First, disease modules are inferred from transcriptomics data. Second, the inferred modules are filtered based on hub regulation resulting in a core set of genes with strong disease association.

The pipeline is built on the previously developed tools MODifieR and ComHub. MODifieR includes eight commonly used methods for disease module inference, all which are integrated in MODalyseR. These methods often predict modules containing hundreds or thousands of genes. To further limit these modules to the genes with the strongest disease association, hubs inferred with ComHub are used to filter the modules based on hub regulation.

ComHub robustly predicts hub regulators by averaging over the predictions by a compendium of gene regulatory network inference methods. MODalyseR includes seven commonly used network inference methods from the ComHub package, with the possibility to also include in-house network predictions. The output of ComHub is the average outdegree of each regulator selecting the TFs with highest degree as hubs. The default value in ComHub is set to 10%, which previously resulted in highest performance (Åkesson *et al.*, 2021), but can be changed in the software by the user.

Interactions between hubs and module genes are determined by overlaying the hubs and module genes on a gene regulatory network. In MODalyseR, a consensus network inferred by averaging over the predictions of the available gene regulatory network inference methods from ComHub is used by default. Module genes, which are regulated by hubs, are kept in the module. A hub should have a significant overrepresentation of interactions with module genes compared to the remaining network for the hub to be used for filtering the module genes, assessed with Fisher’s exact test (odds ratio >2 and *P* < 0.05). This approach results in a smaller module containing more disease significant genes.

MODalyseR includes several options for performing functional and disease analysis of inferred modules. First, gene set enrichment analysis of GO terms, KEGG pathways and disease ontology terms. Second, genes can be labeled with user-chosen terms, such as known biomarkers or protein secretion. Third, modules can be easily exported to Cytoscape for visualization.

### 2.3 Use case

We applied MODalyseR on RNA-seq data of MS white matter lesions with Gene Expression Omnibus (GEO) accession number GSE138614 (Elkjaer *et al.*, 2019). We chose this dataset as we could directly compare the success of our pipeline using active and non-active lesions as positive and negative controls, respectively. The dataset consisted of 71 white matter samples from 10 patients with progressive MS and 25 control white matter samples from 5 donors. The MS samples were from five different white matter areas: 21 Normal appearing white matter (NAWM), 5 remyelinating lesions, 15 active lesions, 14 inactive lesions and 16 chronic active lesions. Differential gene expression analysis for each contrast was acquired from Supplementary Material in GEO, in addition to the raw RNA-seq count data. The count data were normalized with the function *varianceStabilizingTransformation* from the R package DESeq2 (Love *et al.*, 2014) and subsequently quantile normalized using *normalize.quantiles* from the R package preprocessCore (Bolstad, 2019). The R script used for preprocessing can be found in Supplementary File S1.

### 2.4 Chip-seq confirmation of MS-associated targets of IKZF1

We validated the TF IKZF1 as a regulator of MS by calculating the enrichment of SNPs associated with MS in IKZF1 binding regions. The enrichment of MS SNPs were calculated using the function permTest from the R package regioneR (Gel *et al.*, 2016), which performs a permutation test. SNPs significantly associated with MS, using a nominal $P<1\times 10^{-5}$ as we previously did (Hellberg *et al.*, 2016), were obtained from Sawcer *et al.* (2011). We used all SNVs in linkage disequilibrium with the MS-associated SNPs computed using SNiPA (Arnold *et al.*, 2015) (*r*^2^ > 0.8, 1000 Genomes Phase 3 v5 variant set, European population). There were 11 249 SNPs in linkage disequilibrium with the 710 MS SNPs. IKZF1-binding sites from IKZF1 ChIP-seq on human GM12878 were downloaded from the ENCODE portal (Davis *et al.*, 2018) (https://www.encodeproject.org/). We used conservative idr thresholded peaks with identifier ENCFF448JWW. Common SNVs in NCBI dbSNP Build 153 with RegulomeDB rank scores were downloaded from RegulomeDB (Boyle *et al.*, 2012) (https://regulomedb.org/). SNVs and IKZF1 binding regions were annotated with promoter regions using the function annotatePeak from the R package ChIPseeker (Yu *et al.*, 2015).

## 3 Results

### 3.1 MODalyseR

Tools for network medicine has so far most often focused on individual network-based concepts alone and often require the installation of several dependencies making the combinations of multiple different concepts complicated for medicine-oriented labs and others. In addition, many of the state-of-the-art methods take substantial computational power limiting the efficiency of online services for this service. We therefore created MODalyseR as a standalone interactive R Shiny (Chang *et al.*, 2020) web application implemented in the golem (Guyader *et al.*, 2020) framework and shipped as a Docker image that can easily be run on a local machine. The MODalyseR docker image is available at https://hub.docker.com/r/ddeweerd/modalyser and a detailed user guide and installation instructions can be found at https://gustafsson-lab.gitlab.io/MODalyseR/. MODalyseR incorporates eight disease module inference methods previously described in the MODifieR package. In addition, the software includes hub detection from the python package ComHub to infer hub regulators of the disease modules using seven different methods. Both types of methods can easily be run after each other using mRNA expression data and an external network of interest as input. We implemented enrichment analysis for the user to see progress and being able to adjust hyperparameters at the different steps based on functional data.

**Fig. 2.**
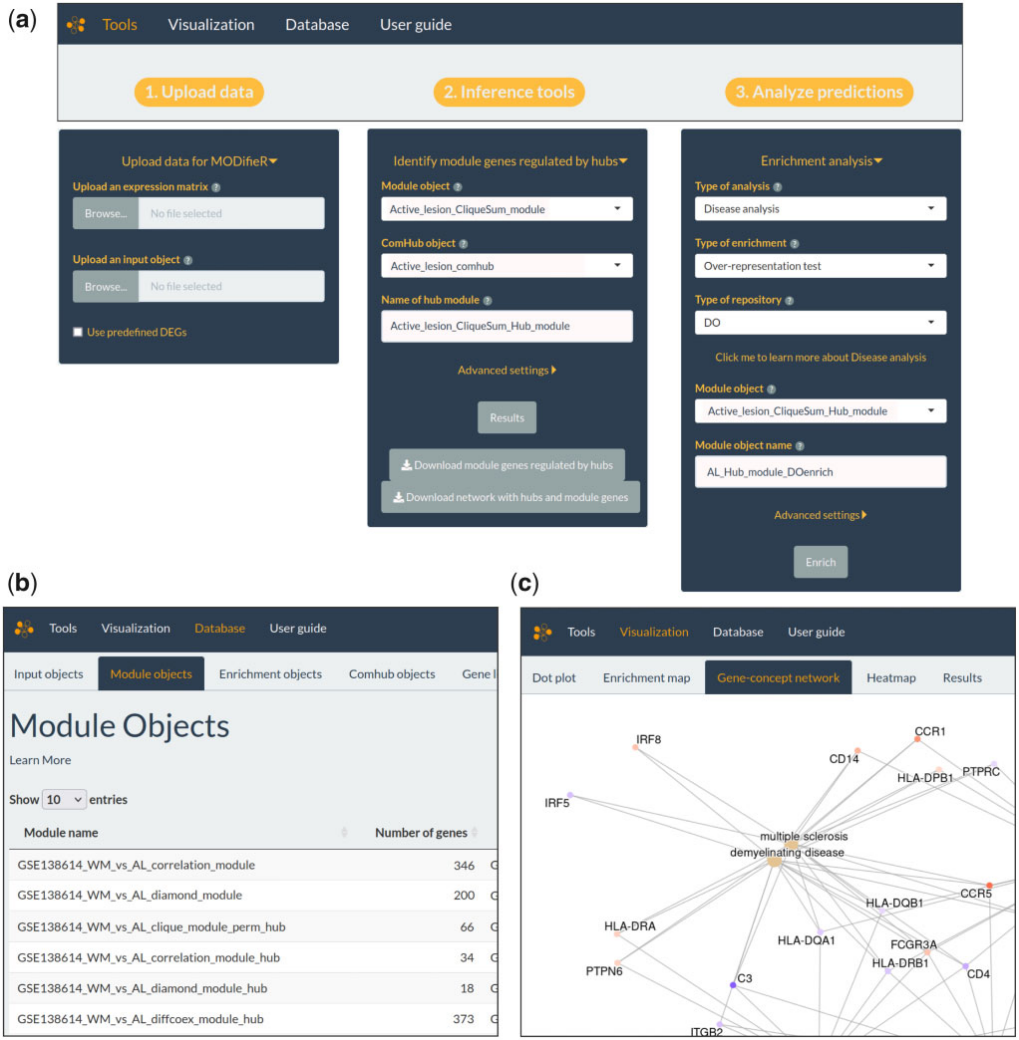
MODalyseR interface. (**a**) Tools, the main page of the application. This page has all the functionality to conduct the analysis, from the upload of input data to module inference, hub detection, filtering of modules by significant hubs to enrichment analysis. (**b**) In the visualization tab, the results of enrichment analysis are inspected. There are several downloadable plots and a searchable jQuery datatable. (**c**) In the database tab all stored data are viewed and curated. Every type of object has its own sub tab with a searchable jQuery datatable

The interface of MODalyseR is divided into three main tabs, namely tools, visualization and database. The tools tab (Fig. 2a) is further divided into three columns of related functions following the general flow (Fig. 1) of the analysis. The first block, 1. Upload data deals with the uploading of all input data. Disease module inference and subsequent hub detection is performed in the second block 2. Inference methods. In the third block 3. Analyze predictions the results can be further scrutinized with for example pathway or GO term enrichment or sent to Cytoscape for more downstream analysis and visualization. Next, the tab called visualization (Fig. 2b) shows results from the functional and disease enrichment analysis in 3. Analyze predictions can be inspected with a set of reactive plots, including a dot plot, enrichment map and gene concept network from the clusterProfiler R package (Yu *et al.*, 2012). In addition, a searchable jQuery datatable from the R package DT (Xie *et al.*, 2021) containing all enriched terms and statistics is available. Lastly, the tab named Database (Fig. 2c) consists of sub tabs with searchable jQuery datatables where all stored objects per category can be viewed and curated. In addition, each of the methods has different hyperparameters that the user can set and rerun enrichment analysis for finetuning in an iterative procedure.

The input for MODalyseR is a set of differentially expressed genes, a normalized RNA-seq count matrix and optional annotation. The input data are wrapped in an input object that is used for all disease module inference methods. Additionally, a protein–protein interaction (PPI) network is required for certain disease module inference methods. MODalyseR offers a built-in function to obtain a STRING PPI (Szklarczyk *et al.*, 2019) network, which can be set using a custom confidence score cutoff. The MODifieR input object can easily be converted to a ComHub object to work with the gene regulatory inference methods. After processing the data in the general workflow as described in Figure 1, the result consists of module objects containing a hub-filtered list of module genes that can be used for enrichment analysis or visualized in Cytoscape with optional annotation added.

### 3.2 Hub-based module filtering revealed IKZF1 as a putative MS-associated TF regulator, which was justified by independent ChIP-seq data

**Fig. 3.**
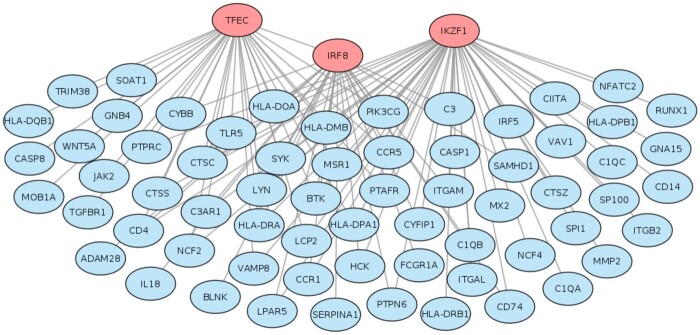
The hubs IKZF1, IRF8 and TFEC (red) were identified as significant regulators of the active lesion CliqueSum module. The module was reduced to 66 genes (blue) with strong enrichment of MS

To demonstrate the applicability of MODalyseR, we applied it to a recent dataset of MS including five types of areas of the white matter lesions, hereafter called active lesion, chronic active lesion, remyelinating lesion, inactive lesion and NAWM (Table 1). The three first represent inflammatory areas and the two last are expected to be more similar to controls. First, we computed modules using default MODalyseR parameters for each of the eight module inference methods across lesions and calculated disease significance (Step 4: Fig. 1). This analysis showed the CliqueSum modules to yield highest significance for MS according to Disease Gene Ontology (DGO), which also is consistent with our previous benchmark study (Badam *et al.*, 2021). The modules covered 1193–1498 genes each and three were enriched for MS, whereof the non-inflammatory NAWM was one. Next, we proceeded with hub filtering, which resulted in empty modules for the inactive lesion and NAWM and modules of size 66, 32 and 38 genes for the active lesions (Fig. 3), chronic active lesions (Supplementary Fig. S1) and remyelinating lesions (Supplementary Fig. S2), respectively. DGO analysis showed these modules to be significantly enriched of MS and three hub TFs were enriched for bindings in these filtered modules, namely IKZF1 (all three lesions), IRF8 (two lesions) and TFEC (one lesion) (Table 1). MS were among the two most enriched diseases for all three modules (Supplementary Tables S1–S3). In addition, these modules were enriched for KEGG pathways, such as Th17 cell differentiation and Th1 and Th2 cell differentiation, which indicates inflammatory activity (Supplementary Tables S4–S6).

**Table 1.** Enrichment of MS for CliqueSum inferred modules before and after hub filtering

	Clique sum module		Hub-filtered clique sum module		
White matter area	Size	MS enrichment	Hub TFs	Size	MS enrichment
Active lesion	1498	$1.07\times 10^{-9}$	IKZF1, IRF8, TFEC	66	$5.23\times 10^{-10}$
Chronic active lesion	1193	0.82	IKZF1, IRF8,	32	$1.8\times 10^{-3}$
Remyelinating lesion	1432	$9.3\times 10^{-5}$	IKZF1	38	$1.33\times 10^{-12}$
Inactive lesion	1336	1	—	—	—
NAWM	1206	$7.6\times 10^{-6}$	—	—	—

**Fig. 4.**
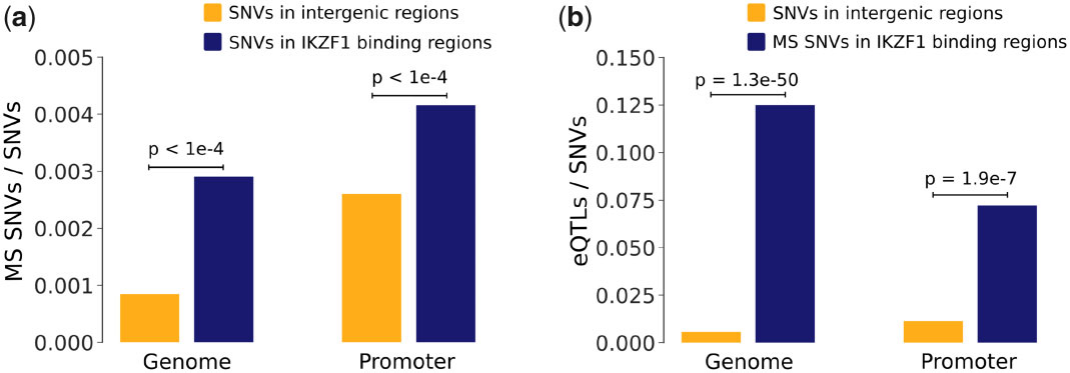
IKZF1-binding regions were enriched for MS SNPs. (**a**) Proportion of MS SNPs among common SNVs in intergenic regions compared to IKZF1-binding sites, both for the genome and promoter regions. IKZF1-binding sites were significantly enriched for MS SNPs evaluated with a permutation test. (**b**) Proportion of eQTLs among SNVs in intergenic regions compared to proportion of eQTLs among MS SNPs in IKZF1-binding sites, both for the genome and promoter regions. MS SNPs in IKZF1-binding sites were significantly enriched for eQTLs evaluated with Fisher’s exact test

As our interactions were derived from mRNA correlations, we next aimed to test if they could act directly between the TF and target promoters. As IKZF1 was consistently identified in all active lesions, we reasoned that it plays a central role in lesions and therefore mapped DNA binding regions from ChIP-seq data of IKZF1 using data from ENCODE and RegulomeDB. Indeed, we found that all binding regions of IKZF1 were generally enriched for MS-associated SNPs both at distal and promoter positions (permutation test $P<10^{-4}$) (Fig. 4a). Lastly, we tested if those SNPs were annotated (using RegulomeDB) to have a downstream effect of the target gene expression (so called eQTLs) and found a highly significant 10- and 7-fold enrichment versus intergenic ($P=10^{-50}$) and promoter regions ($P=2\times 10^{-7}$), respectively (Fig. 4b). Taken together, our use case shows that our implemented two network-based methods can be combined for increased sensitivity and detect direct upstream important regulators.

## 4 Discussion

Disease modules are often used to model complex diseases with the aim to uncover disease biomarkers, which could be used for early diagnosis and personalized treatments. However, existing methods often include many false positive genes in the disease modules, making it difficult to utilize the modules in clinical applications. Studies in the field of network-based disease module identification have been restricted to present new module inference methods and apply modules to explore disease mechanisms with the aim to identify gene candidates associated with the studied disease. While these studies are often delimited to only use PPI networks in combination with some omics data, we propose a novel approach that adds an additional layer of knowledge in terms of regulatory networks, and more specifically regulatory hubs. To our knowledge, it is a unique way to enhance disease modules approach with a regulatory hubs layer, which can accelerate future clinical use, as hubs have gained huge importance in systems medicine and are commonly recognized as potential disease biomarker candidates. With MODalyseR, we do not only introduce a novel scientific approach that integrates regulatory mechanisms within sub-modules of highly interacting genes with shared upstream regulators to identify a core set of genes with strong disease association. We also provide user-friendly software for network-based disease analysis that enables biomedical researchers without computational background to explore disease-related mechanisms, and select interesting biomarker candidates.

MODalyseR implements a pipeline, which reduces modules to the most important genes, which could serve well as potential robust biomarkers. With MODalyseR, we predicted highly MS enriched modules and found IKZF1 as a possible master regulator of MS in white matter lesions. IKZF1 is an important TF for the development of lymphocytes and has previously been connected to leukemia and autoimmune diseases (Hoshino *et al.*, 2017; Marke *et al.*, 2018). Validation of IKZF1 using independent Chip-seq analysis showed a strong significant enrichment of MS-associated SNPs in IKZF1 binding regions, which also were enriched for eQTLs. Hence, the regulatory mechanism of IKZF1 is likely altered in MS affecting downstream genes. This case study shows how MODalyseR can be used to elucidate the regulatory mechanism of complex diseases by both identifying a limited set of disease-associated genes and the main regulators of those genes. There are also other pipelines, such as NeTFactor (Ahsen *et al.*, 2019), which are aimed at predicting regulators of gene expression-based biomarkers. However, NeTFactor requires a set of biomarkers to already exist while MODalyseR is directly applied on RNA-seq data.

Recently, Ruan and Wang (2021) have proposed a network-assisted framework Disease-Specific Network Enhancement Prioritization with the similar aim to use networks for disease-specific gene prioritization. While they use disease omics and methylation enhanced cancer-specific networks, our approach adds an additional layer in terms of network regulatory input that supports gene prioritization related to disease modules. As future works, transfer and multi-task learning could be considered among different organisms, in-line with a recent study (Mignone *et al.*, 2020), that focused on identifying hub regulators for human and mouse by identifying cross-organism similarities and differences. There are several web-based tools available for network analysis, such as NetworkAnalyst 3.0 (Zhou *et al.*, 2019) and eVITTA (Cheng *et al.*, 2021) for transcriptomics analysis and visualization, and GeNECK (Zhang *et al.*, 2019) and shinyBN (Chen *et al.*, 2019) for gene network inference. However, user-friendly web applications for disease module analysis are lacking. MODalyseR is provided as a standalone web application and not as a web server tool. While this demands an installation of docker to use the tool, it was a necessary decision to provide a tool, which can run computationally demanding inference methods. The option to download and run module and network inference methods as a nextflow pipeline, locally or on a cluster, we believe increases the applicability of the software by making it possible to use the tool on large datasets.

In summary, most complex diseases have been analyzed extensively and studied using RNA-seq data. This makes MODalyseR a generally applicable multi-purpose systems medicine tool to be applied on existing disease to generate prior hypothesis testing through easy access to many of the most popular network-based tools using state-of-the-art default parameters. The pipeline also allows simultaneous testing of many methods and datasets in parallel and automatically evaluates the methods best suitable for a particular disease, which we previously saw can vary greatly. Moreover, the advanced user can refine the networks further by integrating other data-sources and further combining the best methods and datasets as we previously shown (Badam *et al.*, 2021). Lastly, the use case stresses how this can be done in practice for in principle any complex disease where RNA-seq data are available.

## Supplementary Material

vbac006_Supplementary_Data

## References

[vbac006-B1] Ahsen M.E. et al (2019) NeTFactor, a framework for identifying transcriptional regulators of gene expression-based biomarkers. Sci. Rep., 9, 12970. 31506535 10.1038/s41598-019-49498-y PMC6737052

[vbac006-B2] Åkesson J. et al (2021) ComHub: community predictions of hubs in gene regulatory networks. BMC Bioinformatics, 22, 58. 33563211 10.1186/s12859-021-03987-y PMC7871572

[vbac006-B3] Arnold M. et al (2015) SNiPA: an interactive, genetic variant-centered annotation browser. Bioinformatics, 31, 1334–1336. 25431330 10.1093/bioinformatics/btu779 PMC4393511

[vbac006-B4] Badam T.V.S. et al (2021) A validated generally applicable approach using the systematic assessment of disease modules by GWAS reveals a multi-omic module strongly associated with risk factors in multiple sclerosis. BMC Genomics, 22, 1–13. 34461822 10.1186/s12864-021-07935-1 PMC8404328

[vbac006-B5] Bader G.D. , Hogue C.W. (2003) An automated method for finding molecular complexes in large protein interaction networks. BMC Bioinformatics, 4, 2. 12525261 10.1186/1471-2105-4-2 PMC149346

[vbac006-B6] Björnsson B. et al; Swedish Digital Twin Consortium. (2019) Digital twins to personalize medicine. Genome Med., 12, 4–13. 31892363 10.1186/s13073-019-0701-3 PMC6938608

[vbac006-B7] Bolstad B. (2019) *preprocessCore: A Collection of Pre-Processing Functions*. R package version 1.46.0. https://github.com/bmbolstad/preprocessCore (5 October 2021, date last accessed).

[vbac006-B8] Boyle A.P. et al (2012) Annotation of functional variation in personal genomes using RegulomeDB. Genome Res., 22, 1790–1797. 22955989 10.1101/gr.137323.112 PMC3431494

[vbac006-B9] Bruhn S. et al (2014) A generally applicable translational strategy identifies S100A4 as a candidate gene in allergy. Sci. Transl. Med., 6, 218ra4. 10.1126/scitranslmed.3007410 PMC4539009 24401939

[vbac006-B10] Chang W. et al (2020) *shiny: Web Application Framework for R*. R package version 1.5.0. https://CRAN.R-project.org/package=shiny (2 October 2021, date last accessed).

[vbac006-B11] Chen J. et al (2019) shinyBN: an online application for interactive Bayesian network inference and visualization. BMC Bioinformatics, 20, 711. 31842743 10.1186/s12859-019-3309-0 PMC6916222

[vbac006-B12] Cheng X. et al (2021) eVITTA: a web-based visualization and inference toolbox for transcriptome analysis. Nucleic Acids Res., 49, W207–W215. 34019643 10.1093/nar/gkab366 PMC8218201

[vbac006-B13] Choobdar S. et al; DREAM Module Identification Challenge Consortium. (2019) Assessment of network module identification across complex diseases. Nat. Methods, 16, 843–852. 31471613 10.1038/s41592-019-0509-5 PMC6719725

[vbac006-B14] Cirillo D. , Valencia A. (2019) Big data analytics for personalized medicine. Curr. Opin. Biotechnol., 58, 161–167. 30965188 10.1016/j.copbio.2019.03.004

[vbac006-B15] Davis C.A. et al (2018) The Encyclopedia of DNA elements (ENCODE): data portal update. Nucleic Acids Res., 46, D794–D801. 29126249 10.1093/nar/gkx1081 PMC5753278

[vbac006-B16] de Weerd H.A. et al (2020) MODifieR: an ensemble R package for inference of disease modules from transcriptomics networks. Bioinformatics, 36, 3918–3919. 32271876 10.1093/bioinformatics/btaa235

[vbac006-B17] Di Tommaso P. et al (2017) Nextflow enables reproducible computational workflows. Nat. Biotechnol., 35, 316–319. 28398311 10.1038/nbt.3820

[vbac006-B18] Elkjaer M.L. et al (2019) Molecular signature of different lesion types in the brain white matter of patients with progressive multiple sclerosis. Acta Neuropathol. Commun., 7, 205–217. 31829262 10.1186/s40478-019-0855-7 PMC6907342

[vbac006-B19] Gel B. et al (2016) regioneR: an R/Bioconductor package for the association analysis of genomic regions based on permutation tests. Bioinformatics, 32, 289–291. 26424858 10.1093/bioinformatics/btv562 PMC4708104

[vbac006-B20] Ghiassian S.D. et al (2015) A DIseAse MOdule Detection (DIAMOnD) algorithm derived from a systematic analysis of connectivity patterns of disease proteins in the human interactome. PLoS Comput. Biol., 11, e1004120. 25853560 10.1371/journal.pcbi.1004120 PMC4390154

[vbac006-B21] Gligorijević V. et al (2016) Integrative methods for analyzing big data in precision medicine. Proteomics, 16, 741–758. 26677817 10.1002/pmic.201500396

[vbac006-B22] Gustafsson M. et al (2014a) Integrated genomic and prospective clinical studies show the importance of modular pleiotropy for disease susceptibility, diagnosis and treatment. Genome Med., 6, 17. 24571673 10.1186/gm534 PMC4064311

[vbac006-B23] Gustafsson M. et al (2014b) Modules, networks and systems medicine for understanding disease and aiding diagnosis. Genome Med., 6, 82. 25473422 10.1186/s13073-014-0082-6 PMC4254417

[vbac006-B24] Gustafsson M. et al (2015) A validated gene regulatory network and GWAS identifies early regulators of T cell-associated diseases. Sci. Transl. Med., 7, 313ra178. 10.1126/scitranslmed.aad2722 26560356

[vbac006-B25] Guyader V. et al (2020) *golem: A Framework for Robust Shiny Applications*. R package version 0.2.1. https://CRAN.R-project.org/package=golem (1 October 2021, date last accessed).

[vbac006-B26] Hellberg S. et al (2016) Dynamic response genes in CD4+ T cells reveal a network of interactive proteins that classifies disease activity in multiple sclerosis. Cell Rep., 16, 2928–2939. 27626663 10.1016/j.celrep.2016.08.036

[vbac006-B27] Hoshino A. et al (2017) Abnormal hematopoiesis and autoimmunity in human subjects with germline IKZF1 mutations. J Allergy Clin. Immunol., 140, 223–231. 27939403 10.1016/j.jaci.2016.09.029

[vbac006-B28] Kurtzer G.M. et al (2017) Singularity: scientific containers for mobility of compute. PLoS One, 12, e0177459. 28494014 10.1371/journal.pone.0177459 PMC5426675

[vbac006-B29] Langfelder P. , Horvath S. (2008) WGCNA: an R package for weighted correlation network analysis. BMC Bioinformatics, 9, 559. 19114008 10.1186/1471-2105-9-559 PMC2631488

[vbac006-B30] Langfelder P. , Horvath S. (2012) Fast R functions for robust correlations and hierarchical clustering. J. Stat. Softw., 46, i11. http://www.ncbi.nlm.nih.gov/pubmed/23050260{\%}0Ahttp://www.pubmedcentral.nih.gov/articlerender.fcgi?artid=PMC3465711. 23050260 PMC3465711

[vbac006-B31] Lefebvre C. et al (2010) A human B-cell interactome identifies MYB and FOXM1 as master regulators of proliferation in germinal centers. Mol. Syst. Biol., 6, 377. 20531406 10.1038/msb.2010.31 PMC2913282

[vbac006-B32] Liu Y. et al (2019) Identification of hub genes and key pathways associated with bipolar disorder based on weighted gene co-expression network analysis. Front. Physiol., 10, 1081. 31481902 10.3389/fphys.2019.01081 PMC6710482

[vbac006-B33] Love M.I. et al (2014) Moderated estimation of fold change and dispersion for RNA-seq data with DESeq2. Genome Biol., 15, 550. 25516281 10.1186/s13059-014-0550-8 PMC4302049

[vbac006-B34] Malod-Dognin N. et al (2018) Precision medicine - a promising, yet challenging road lies ahead. Curr. Opin. Syst. Biol., 7, 1–7.

[vbac006-B35] Marke R. et al (2018) The many faces of IKZF1 in B-cell precursor acute lymphoblastic leukemia. Haematologica, 103, 565–574. 29519871 10.3324/haematol.2017.185603 PMC5865415

[vbac006-B36] Menche J. et al (2015) Disease networks. Uncovering disease-disease relationships through the incomplete interactome. Science, 347, 1257601. 25700523 10.1126/science.1257601 PMC4435741

[vbac006-B37] Mignone P. et al (2020) Multi-task learning for the simultaneous reconstruction of the human and mouse gene regulatory networks. Sci. Rep., 10, 22295. 33339842 10.1038/s41598-020-78033-7 PMC7749184

[vbac006-B39] Müller K. et al (2020) *RSQLite: ‘SQLite’ Interface for R*. R package version 2.2.1. https://CRAN.R-project.org/package=RSQLite (9 October 2021, date last accessed).

[vbac006-B40] Olsson T. et al (2017) Interactions between genetic, lifestyle and environmental risk factors for multiple sclerosis. Nat. Rev. Neurol., 13, 25–36. 27934854 10.1038/nrneurol.2016.187

[vbac006-B41] Ono K. et al (2015) CyREST: turbocharging cytoscape access for external tools via a RESTful API [version 1; referees: 2 approved]. F1000Res., 4, 478. 26672762 10.12688/f1000research.6767.1 PMC4670004

[vbac006-B42] Ruan P. , Wang S. (2021) DiSNEP: a disease-specific gene network enhancement to improve prioritizing candidate disease genes. Brief Bioinform., 22, bbaa241. 33064143 10.1093/bib/bbaa241

[vbac006-B43] Sawcer S. et al; Wellcome Trust Case Control Consortium 2. (2011) Genetic risk and a primary role for cell-mediated immune mechanisms in multiple sclerosis. Nature, 476, 214–219. 21833088 10.1038/nature10251 PMC3182531

[vbac006-B44] Shannon P. et al (2003) Cytoscape: a software environment for integrated models of biomolecular interaction networks. Genome Res., 13, 2498–2504. 14597658 10.1101/gr.1239303 PMC403769

[vbac006-B45] Szklarczyk D. et al (2019) STRING v11: protein-protein association networks with increased coverage, supporting functional discovery in genome-wide experimental datasets. Nucleic Acids Res., 47, D607–D613. 30476243 10.1093/nar/gky1131 PMC6323986

[vbac006-B38] The Multiple Sclerosis International Federation Atlas of MS, 3rd ed, September, 2020, https://www.atlasofms.org (21 September 2021, date last accessed)

[vbac006-B46] Vlaic S. et al (2018) ModuleDiscoverer: identification of regulatory modules in protein-protein interaction networks. Sci. Rep., 8, 433. 29323246 10.1038/s41598-017-18370-2 PMC5764996

[vbac006-B47] Xie Y. et al (2021) *DT: A Wrapper of the JavaScript Library ‘DataTables’*. R package version 0.17. https://CRAN.R-project.org/package=DT (9 October 2021, date last accessed).

[vbac006-B48] Yu G. et al (2012) clusterProfiler: an R package for comparing biological themes among gene clusters. OMICS, 16, 284–287. 22455463 10.1089/omi.2011.0118 PMC3339379

[vbac006-B49] Yu G. et al (2015) ChIPseeker: an R/Bioconductor package for ChIP peak annotation, comparison and visualization. Bioinformatics, 31, 2382–2383. 25765347 10.1093/bioinformatics/btv145

[vbac006-B50] Zhang M. et al (2019) GeNeCK: a web server for gene network construction and visualization. BMC Bioinformatics, 20, 12. 30616521 10.1186/s12859-018-2560-0 PMC6323745

[vbac006-B51] Zhou G. et al (2019) NetworkAnalyst 3.0: a visual analytics platform for comprehensive gene expression profiling and meta-analysis. Nucleic Acids Res., 47, W234–W241. 30931480 10.1093/nar/gkz240 PMC6602507

