# Supplementary material for: MODalyseR—a novel software for inference of disease module hub regulators identified a putative multiple sclerosis regulator supported by independent eQTL data
Source: Bioinform Adv. 2022 Jan 25;2(1):vbac006. doi: 10.1093/bioadv/vbac006 (PMC9710626; doi:10.1093/bioadv/vbac006)
Supplement: vbac006_Supplementary_Data [file vbac006_supplementary_data.zip › S1_use_case_preprocessing.pdf]

MODalyseR use case preprocessing

Dirk de Weerd, Julia Åkesson

```r
library(tidyverse)
library(org.Hs.eg.db)
library(data.table)
library(magrittr)
library(HGNChelper)

read_annotation <- function(deg_data, gene_map){
  suppressWarnings(deg_data %>%
    tidyr::separate(., Label, c("ensemble", "symbol"), ";", F) %>%
    mutate(symbol = replace(symbol, symbol == "NA", NA)) %>%
    mutate(., symbol = HGNChelper::checkGeneSymbols(symbol, map = gene_map)[,3]) %>%
    drop_na(., symbol))
}

normalize_counts <- function(count_matrix){
  DESeq2::varianceStabilizingTransformation(count_matrix) %>%
    preprocessCore::normalize.quantiles(., copy = TRUE) %>%
    set_colnames(.,  make.names(colnames(count_matrix), unique = T)) %>%
    set_rownames(., rownames(count_matrix))
}

filter_counts <- function(deg_genes, counts, labels){
  counts[deg_genes$Label, labels]
}

remove_rows <- function(data, rows){
  data[rows,]
}

build_diff_genes <- function(edge_table){
  dplyr::select(edge_table, entrez, FDR)
}

write_data <- function(diff_genes_table, count_table, dataset_name, edgeR_table){
  dir.create(dataset_name)
  write.table(diff_genes_table, paste0(dataset_name, "/diff_genes.csv"))
  write.table(count_table, paste0(dataset_name, "/count_table.csv"))
  write.table(edgeR_table, paste0(dataset_name, "/edgeR_table.csv"))
}

append_entrez <- function(deg_genes, mapping){
  deg_genes %>% mutate(., entrez = unname(mapping[.$symbol])) %>%
    distinct(., symbol, .keep_all = T)
}


gene_map <- getCurrentHumanMap()
```

```r
load(url("https://github.com/frischt/supplementary_files/blob/master/edgeRScriptData.RData?raw=true"))


#Download DEGs and separate  ensembl and gene symbols
AL <- data.table::fread(
  "https://ftp.ncbi.nlm.nih.gov/geo/series/GSE138nnn/GSE138614/suppl/GSE138614_WM_vs_AL.txt.gz") %>%
  read_annotation(., gene_map = gene_map)
CA <- data.table::fread(
  "https://ftp.ncbi.nlm.nih.gov/geo/series/GSE138nnn/GSE138614/suppl/GSE138614_WM_vs_CA.txt.gz") %>%
  read_annotation(., gene_map = gene_map)
IL <- data.table::fread(
  "https://ftp.ncbi.nlm.nih.gov/geo/series/GSE138nnn/GSE138614/suppl/GSE138614_WM_vs_IL.txt.gz") %>%
  read_annotation(., gene_map = gene_map)
NAWM <- data.table::fread(
  "https://ftp.ncbi.nlm.nih.gov/geo/series/GSE138nnn/GSE138614/suppl/GSE138614_WM_vs_NAWM.txt.gz") %>%
  read_annotation(., gene_map = gene_map)
RL <- data.table::fread(
  "https://ftp.ncbi.nlm.nih.gov/geo/series/GSE138nnn/GSE138614/suppl/GSE138614_WM_vs_RL.txt.gz") %>%
  read_annotation(., gene_map = gene_map)

deg_genes <- list(AL, CA, IL, NAWM, RL) %>%
  set_names(., c("AL", "CA", "IL", "NAWM", "RL"))

symbols <- as.list(org.Hs.egSYMBOL2EG) %>%
  map_chr(., 1)

deg_genes <- lapply(deg_genes, append_entrez, mapping = symbols)

counts <- dataMatrix

#Controls
controls <- which(labeling$Lesion.type..1. == "WM")
colnames(counts)[controls] <- "control"
#Normal appearing white matter
nawm <- which(labeling$Lesion.type..1. == "NAWM")
colnames(counts)[nawm] <- "nawm"
#Active lesions
al <- which(labeling$Lesion.type..1. == "AL")
colnames(counts)[al] <- "al"
#Remyelinating
rl <- which(labeling$Lesion.type..1. == "RL")
colnames(counts)[rl] <- "rl"
#Chronic lesions
ca <- which(labeling$Lesion.type..1. == "CA")
colnames(counts)[ca] <- "ca"
#Inactive lesions
il <- which(labeling$Lesion.type..1. == "IL")
colnames(counts)[il] <- "il"

all_ms <- list(al, ca, il, nawm, rl) %>%
  set_names(., c("AL", "CA", "IL", "NAWM", "RL"))

counts_per_contrast <- list()

for (i in 1:length(deg_genes)){

  counts_per_contrast[[i]] <- filter_counts(deg_genes = deg_genes[[i]],
                                             counts = counts,
```

```
                                             labels = c(all_ms[[i]], controls))

}

names(counts_per_contrast) <- names(deg_genes)

for (i in 1:length(counts_per_contrast)){
  rows <- !is.na(deg_genes[[i]]$entrez)
  rownames(counts_per_contrast[[i]]) <- deg_genes[[i]]$entrez
  counts_per_contrast[[i]] <- remove_rows(counts_per_contrast[[i]], rows)
  deg_genes[[i]] <- remove_rows(deg_genes[[i]], rows)

}

diff_genes <- lapply(deg_genes, build_diff_genes) %>%
  set_names(., names(deg_genes))

normalized_counts <- lapply(counts_per_contrast, normalize_counts) %>%
  set_names(., names(counts_per_contrast))

for (i in 1:length(diff_genes)){
  write_data(diff_genes[[i]],
             normalized_counts[[i]],
             names(diff_genes)[i],
             deg_genes[[i]])
}
```
