# Supplementary material for: MODalyseR—a novel software for inference of disease module hub regulators identified a putative multiple sclerosis regulator supported by independent eQTL data
Source: Bioinform Adv. 2022 Jan 25;2(1):vbac006. doi: 10.1093/bioadv/vbac006 (PMC9710626; doi:10.1093/bioadv/vbac006)
Supplement: vbac006_Supplementary_Data [file vbac006_supplementary_data.zip › S2_figures_and_tables.pdf]

Supplementary material 2

MODalyseR - a novel software for inference of disease module hub regulators identified a putative Multiple Sclerosis regulator supported by independent eQTL data

Hendrik A. de Weerd, Julia Åkesson, Dimitri Guala, Mika Gustafsson, Zelmina Lubovac-Pilav

List of figures:

Figure S1 Hub-filtered Clique Sum module for chronic active lesions……………………... 1
Figure S2 Hub-filtered Clique Sum module for remyelinating lesions……………………... 1

List of tables:

Table S1: Disease ontology enrichment analysis of hub-filtered Clique Sum module for active lesions……………………………………………………………………………..2
Table S2: disease ontology enrichment analysis of hub-filtered Clique Sum module for chronic active lesions………..……………………………………………………………2
Table S3: disease ontology enrichment analysis of hub-filtered Clique Sum module for remyelinating lesions………..……………………………………………………………3
Table S4: Kegg pathway enrichment analysis of hub-filtered Clique Sum module for active lesions……..…………………………………………………………………... 4
Table S5: Kegg pathway enrichment analysis of hub-filtered Clique Sum module for chronic active lesions………..………………………………………………………… 5
Table S6: Kegg pathway enrichment analysis of hub-filtered Clique Sum module for remyelinating lesions………..…………………………………………………………..6

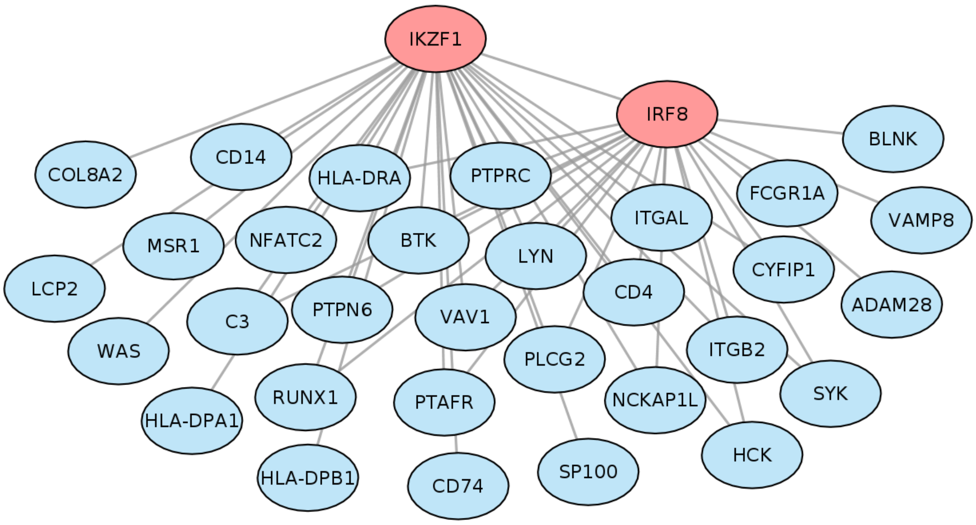

Figure S1: *Hub-filtered Clique Sum module for chronic active lesions. The hubs IKZF1 and IRF8 were identified as significant regulators of the chronic active lesion CliqueSum module. The module was reduced to 32 genes (blue) with strong enrichment of MS.*

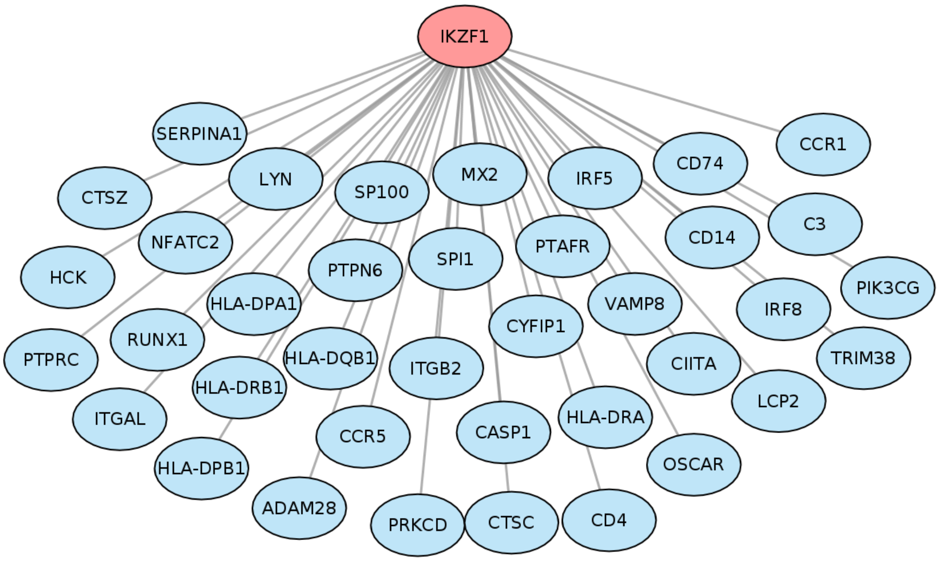

Figure S2: *Hub-filtered Clique Sum module for remyelinating lesions. The hubs IKZF1 identified as a significant regulator of remyelinating lesion CliqueSum module. The module was reduced to 38 genes (blue) with strong enrichment of MS.*

Table S1: *Disease gene ontology enrichment analysis of hub-filtered Clique Sum module for active lesions. The top 20 enriched diseases (p.adjust < 0.05) are shown.*

Description	GeneRatio	p.adjust
demyelinating disease	17/59	5.39e-11
multiple sclerosis	16/59	5.23e-10
rheumatoid arthritis	24/59	2.62e-9
primary immunodeficiency disease	16/59	4.28e-9
bacterial infectious disease	15/59	4.70E-06
lung disease	19/59	1.41E-05
lower respiratory tract disease	19/59	1.93E-05
hepatitis	17/59	7.35E-05
myeloid leukemia	10/59	0.000217
multiple myeloma	13/59	0.000220
autoimmune disease of the nervous system	7/59	0.000257
chronic obstructive pulmonary disease	12/59	0.000279
hepatitis C	12/59	0.000618
hepatitis B	11/59	0.000625
lymphoblastic leukemia	15/59	0.000785
obstructive lung disease	13/59	0.00132
hematopoietic system disease	16/59	0.00133
Human immunodeficiency virus infectious disease	10/59	0.00152

Table S2: *Disease gene ontology enrichment analysis of hub-filtered Clique Sum module for chronic active lesions. The top 20 enriched diseases (p.adjust < 0.05) are shown.*

Description	GeneRatio	p.adjust
demyelinating disease	9/30	1.51E-05
multiple sclerosis	8/30	0.000185
hematopoietic system disease	10/30	0.00770
lymphoblastic leukemia	9/30	0.0129
autoimmune disease of the nervous system	4/30	0.0285
multiple myeloma	7/30	0.0392
bacterial infectious disease	7/30	0.0455

Table S3: *Disease gene ontology enrichment analysis of hub-filtered Clique Sum module for remyelinating lesions. The top 20 enriched diseases (p.adjust < 0.05) are shown.*

Description	GeneRatio	p.adjust
demyelinating disease	16/36	7.03E-14
multiple sclerosis	15/36	1.33E-12
rheumatoid arthritis	15/36	1.43E-05
bacterial infectious disease	11/36	4.45E-05
autoimmune disease of the nervous system	6/36	0.000167
primary immunodeficiency disease	9/36	0.000298
multiple myeloma	10/36	0.000366
chronic leukemia	9/36	0.000466
hepatitis	12/36	0.000691
Human immunodeficiency virus infectious disease	8/36	0.00136
hepatitis C	9/36	0.00167
myeloma	10/36	0.00243
bone marrow cancer	10/36	0.00273
hepatitis B	8/36	0.00348
tuberculosis	7/36	0.00608
liver cirrhosis	8/36	0.00851
endocrine system disease	10/36	0.0119
primary bacterial infectious disease	8/36	0.0119
lymphoblastic leukemia	10/36	0.0143

Table S4: *Kegg pathway enrichment analysis of hub-filtered Clique Sum module for active lesions. Only pathways belonging to the category organismal systems are shown.*

ID	Description	GeneRatio	p.adjust	geneID
hsa04612	Antigen processing and presentation	11/64	4.71E-10	CTSS/HLA-DMB/HLA-DOA/HLA-DPA1/HLA-DPB1/HLA-DQB1/HLA-DRA/HLA-DRB1/CIITA/CD4/CD74
hsa04659	Th17 cell differentiation	12/64	5.96E-10	HLA-DMB/HLA-DOA/HLA-DPA1/HLA-DPB1/HLA-DQB1/HLA-DRA/HLA-DRB1/JAK2/NFATC2/TGFBR1/RUNX1/CD4
hsa04640	Hematopoietic cell lineage	11/64	3.73E-09	FCGR1A/HLA-DMB/HLA-DOA/HLA-DPA1/HLA-DPB1/HLA-DQB1/HLA-DRA/HLA-DRB1/ITGAM/CD4/CD14
hsa04658	Th1 and Th2 cell differentiation	10/64	2.12E-08	HLA-DMB/HLA-DOA/HLA-DPA1/HLA-DPB1/HLA-DQB1/HLA-DRA/HLA-DRB1/JAK2/NFATC2/CD4
hsa04380	Osteoclast differentiation	10/64	3.13E-07	FCGR1A/BLNK/LCP2/NCF2/NCF4/NFATC2/SPI1/SYK/BTK/TGFBR1
hsa04672	Intestinal immune network for IgA production	7/64	5.45E-07	HLA-DMB/HLA-DOA/HLA-DPA1/HLA-DPB1/HLA-DQB1/HLA-DRA/HLA-DRB1
hsa04610	Complement and coagulation cascades	8/64	1.55E-06	ITGAM/ITGB2/SERPINA1/C1QA/C1QB/C1QC/C3/C3AR1
hsa04613	Neutrophil extracellular trap formation	10/64	1E-05	CYBB/FCGR1A/ITGAL/ITGAM/ITGB2/NCF2/NCF4/SYK/C3/CASP1
hsa04670	Leukocyte transendothelial migration	8/64	1.34E-05	CYBB/ITGAL/ITGAM/ITGB2/MMP2/NCF2/NCF4/VAV1
hsa04662	B cell receptor signaling pathway	7/64	1.54E-05	BLNK/LYN/NFATC2/PTPN6/SYK/BTK/VAV1
hsa04650	Natural killer cell mediated cytotoxicity	7/64	0.000305	ITGAL/ITGB2/LCP2/NFATC2/PTPN6/SYK/VAV1
hsa04666	Fc gamma R-mediated phagocytosis	6/64	0.000437	FCGR1A/HCK/LYN/PTPRC/SYK/VAV1
hsa04062	Chemokine signaling pathway	8/64	0.00047	CCR1/CCR5/HCK/JAK2/LYN/PIK3CG/GNB4/VAV1
hsa04660	T cell receptor signaling pathway	6/64	0.000597	LCP2/NFATC2/PTPN6/PTPRC/VAV1/CD4
hsa04664	Fc epsilon RI signaling pathway	5/64	0.000668	LCP2/LYN/SYK/BTK/VAV1
hsa04611	Platelet activation	6/64	0.00145	LCP2/LYN/PIK3CG/SYK/BTK/VAMP8
hsa04620	Toll-like receptor signaling pathway	4/64	0.0239	IRF5/TLR5/CASP8/CD14
hsa04625	C-type lectin receptor signaling pathway	4/64	0.0239	NFATC2/SYK/CASP1/CASP8
hsa04926	Relaxin signaling pathway	4/64	0.0477	GNA15/MMP2/GNB4/TGFBR1

Table S5: *Kegg pathway enrichment analysis of hub-filtered Clique Sum module for chronic active lesions. Only pathways belonging to the category organismal systems are shown.*

**ID**	**Description**	**GeneRatio**	**p.adjust**	**geneID**
hsa04662	B cell receptor signaling pathway	8/31	5.2E-08	BLNK/LYN/NFATC2/PLCG2/PTPN6/SYK/BTK/VAV1
hsa04666	Fc gamma R-mediated phagocytosis	8/31	6.77E-08	FCGR1A/HCK/LYN/PLCG2/PTPRC/SYK/VAV1/WAS
hsa04650	Natural killer cell mediated cytotoxicity	8/31	3.71E-07	ITGAL/ITGB2/LCP2/NFATC2/PLCG2/PTPN6/SYK/VAV1
hsa04664	Fc epsilon RI signaling pathway	6/31	1.99E-06	LCP2/LYN/PLCG2/SYK/BTK/VAV1
hsa04380	Osteoclast differentiation	7/31	4.1E-06	FCGR1A/BLNK/LCP2/NFATC2/PLCG2/SYK/BTK
hsa04640	Hematopoietic cell lineage	6/31	1.4E-05	FCGR1A/HLA-DPA1/HLA-DPB1/HLA-DRA/CD4/CD14
hsa04660	T cell receptor signaling pathway	6/31	1.61E-05	LCP2/NFATC2/PTPN6/PTPRC/VAV1/CD4
hsa04659	Th17 cell differentiation	6/31	1.77E-05	HLA-DPA1/HLA-DPB1/HLA-DRA/NFATC2/RUNX1/CD4
hsa04611	Platelet activation	6/31	3.7E-05	LCP2/LYN/PLCG2/SYK/BTK/VAMP8
hsa04612	Antigen processing and presentation	5/31	5.79E-05	HLA-DPA1/HLA-DPB1/HLA-DRA/CD4/CD74
hsa04658	Th1 and Th2 cell differentiation	5/31	0.000111	HLA-DPA1/HLA-DPB1/HLA-DRA/NFATC2/CD4
hsa04613	Neutrophil extracellular trap formation	6/31	0.00031	FCGR1A/ITGAL/ITGB2/PLCG2/SYK/C3
hsa04062	Chemokine signaling pathway	5/31	0.00234	HCK/LYN/PLCG2/VAV1/WAS
hsa04672	Intestinal immune network for IgA production	3/31	0.00258	HLA-DPA1/HLA-DPB1/HLA-DRA
hsa04670	Leukocyte transendothelial migration	4/31	0.00264	ITGAL/ITGB2/PLCG2/VAV1
hsa04625	C-type lectin receptor signaling pathway	3/31	0.0172	NFATC2/PLCG2/SYK

Table S6: *Kegg pathway enrichment analysis of hub-filtered Clique Sum module for remyelinating lesions. Only pathways belonging to the category organismal systems are shown.*

ID	Description	GeneRatio	p.adjust	geneID
hsa04612	Antigen processing and presentation	8/36	4.46E-08	HLA-DPA1/HLA-DPB1/HLA-DQB1/HLA-DRA/HLA-DRB1/CIITA/CD4/CD74
hsa04659	Th17 cell differentiation	8/36	2.43E-07	HLA-DPA1/HLA-DPB1/HLA-DQB1/HLA-DRA/HLA-DRB1/NFATC2/RUNX1/CD4
hsa04658	Th1 and Th2 cell differentiation	7/36	1.3E-06	HLA-DPA1/HLA-DPB1/HLA-DQB1/HLA-DRA/HLA-DRB1/NFATC2/CD4
hsa04640	Hematopoietic cell lineage	7/36	1.7E-06	HLA-DPA1/HLA-DPB1/HLA-DQB1/HLA-DRA/HLA-DRB1/CD4/CD14
hsa04672	Intestinal immune network for IgA production	5/36	1.22E-05	HLA-DPA1/HLA-DPB1/HLA-DQB1/HLA-DRA/HLA-DRB1
hsa04660	T cell receptor signaling pathway	5/36	0.00036	LCP2/NFATC2/PTPN6/PTPRC/CD4
hsa04062	Chemokine signaling pathway	6/36	0.000678	CCR1/CCR5/HCK/LYN/PIK3CG/PRKCD
hsa04650	Natural killer cell mediated cytotoxicity	5/36	0.000958	ITGAL/ITGB2/LCP2/NFATC2/PTPN6
hsa04666	Fc gamma R-mediated phagocytosis	4/36	0.00295	HCK/LYN/PRKCD/PTPRC
hsa04611	Platelet activation	4/36	0.00688	LCP2/LYN/PIK3CG/VAMP8
hsa04380	Osteoclast differentiation	4/36	0.0075	OSCAR/LCP2/NFATC2/SPI1
hsa04662	B cell receptor signaling pathway	3/36	0.0159	LYN/NFATC2/PTPN6
hsa04610	Complement and coagulation cascades	3/36	0.0171	ITGB2/SERPINA1/C3
hsa04613	Neutrophil extracellular trap formation	4/36	0.0252	ITGAL/ITGB2/C3/CASP1
hsa04625	C-type lectin receptor signaling pathway	3/36	0.0277	NFATC2/PRKCD/CASP1
